# Extracts from Microalga *Chlorella sorokiniana* Exert an Anti-Proliferative Effect and Modulate Cytokines in Sheep Peripheral Blood Mononuclear Cells

**DOI:** 10.3390/ani9020045

**Published:** 2019-01-30

**Authors:** Maria Giovanna Ciliberti, Marzia Albenzio, Matteo Francavilla, Gianluca Neglia, Luigi Esposito, Mariangela Caroprese

**Affiliations:** 1 Department of the Sciences of Agriculture, Food and Environment, University of Foggia, Via Napoli 25, 71121 Foggia, Italy; maria.ciliberti@unifg.it (M.G.C.); marzia.albenzio@unifg.it (M.A.); matteo.francavilla@unifg.it (M.F.); 2 Department of Veterinary Medicine and Animal Production, Federico II University, V. F. Delpino 1, 80137 Naples, Italy; neglia@unina.it (G.N.); luigespo@unina.it (L.E.)

**Keywords:** cytokine, sheep, microalga, proliferation, phytosterol

## Abstract

**Simple Summary:**

In the last several years, the animal production sector has been very concerned about the overuse of antibiotics and the concomitant increase in antimicrobial resistance. The objective of this paper was the evaluation of the immune properties of different extracts isolated from *Chlorella sorokiniana* applied to sheep peripheral blood mononuclear cells (PBMCs). In particular, the effects of the unsaponified fraction (UP), the acetylated unsaponified fraction (AUP), and the total lipid fraction (TL) at two concentrations (0.4 and 0.8 mg/mL) on the proliferative response of sheep cells and their cytokine secretion were tested. All the extracts showed an inhibitory effect on cell proliferation, particularly in the presence of the UP fraction at a 0.4 mg/mL concentration. As regards cytokine production, the UP extract tested at a 0.8 mg/mL concentration increased interleukin(IL)-10 production, whereas the TL fraction at 0.4 mg/mL showed a cytokine profile characterized by an increase in both IL-10 secretion and the anti-inflammatory cytokine IL-6, and, to a lesser extent, IL-1β secretions, which are proinflammatory cytokines. These results supported the hypothesis that extracts from *Chlorella sorokiniana* could be considered useful ingredients to be integrated into animal feed with the aim to control immune responses during inflammation and minimize the use of antibiotics.

**Abstract:**

The objective of this experiment was to study the effects of the unsaponified fraction (UP), the acetylated unsaponified fraction (AUP), and the total lipid fraction (TL) extracted and purified from *Chlorella sorokiniana* (CS) on the proliferation and cytokine profile of sheep peripheral blood mononuclear cells (PBMCs). Cells were cultured with 0.4 mg/mL and 0.8 mg/mL concentrations of each extract (UP, AUP, and TL fractions) and activated with 5 μg/mL concanavalin A (ConA) and 1 μg/mL lipopolysaccharide (LPS) at 37 °C for 24 h. PBMCs cultured with ConA and LPS represented the stimulated cells (SC), and PBMCs without ConA and LPS represented the unstimulated cells (USC). Cell-free supernatants were collected to determine IL-10, IL-1β, and IL-6 secretions; on cells, measurement of proliferation was performed. All the extracts tested significantly decreased the cell proliferation; in particular, the UP fraction at 0.4 mg/mL showed the lowest proliferative response. Furthermore, at 0.8 mg/mL, the UP fraction enhanced IL-10 secretion. On the contrary, the TL fraction at 0.4 mg/mL induced an increase in IL-10, IL-6, and, to a lesser extent, IL-1β secretions by cells. The AUP fraction did not change cytokine secretion. The results demonstrated that CS extracts could be useful ingredients in animal feed in order to minimize the use of antibiotics by modulating cell proliferation and cytokine response.

## 1. Introduction

Marine microalgae improve animal metabolism and response to stressors at different levels [1]. The quality of animal feed strongly relates to animal health [2]; consequently, natural substances such as extracts from microalgae can be used as an ingredient in feed in order to improve an animal’s health status. The good health status of animals represents one of the three pillars (along with human and environmental health) of the current concept of “One Health”. 

Several species of microalgae are rich in well-balanced nutrients including carbohydrates, proteins, lipids, and vitamins [2]. All of these components contribute to the use of microalgae as a complete food containing all of the necessary ingredients to promote human health [3]. A chemoprevention role of extracts from microalgae such as carotenoids, fatty acids, glycolipids, polysaccharides, and proteins has been studied due to their suppressive action on cell proliferation, induction of apoptosis, stimulation of antimetastatic and angiogenic responses, and increase in antioxidant and anti-inflammatory activity [4]. The most popular microalgae are *Spirulina* and *Chlorella* due to their high protein content and nutritional value [5,6]. Of the *Chorella* strains, *Chlorella sorokinana* (CS) is the most suitable source of omega (ω)-3 and ω-6 polyunsaturated fatty acids (PUFA), which are mainly extracted using biorefinery-based production methods [7,8,9].

Previous experiments in a sheep model demonstrated that phytosterols extracted and purified from *Dunaliella tertiolecta* could exert an immunomodulatory effect by reducing cellular proliferation during the postpartum period [10] and could modify the peripheral blood mononuclear cells’ (PBMCs) cytokine profile [11]. The control of immune responses to non-infectious stressors—in particular, during the first days postpartum when sheep experience an inflammatory state, or, on the contrary, during immune depression—by feed enrichment with functional molecules could be a suitable strategy to combat antibiotic overuse and reduce antimicrobial resistance.

This experiment aimed at studying the in vitro effects of the unsaponified fraction (UP), the acetylated unsaponified fraction (AUP), and the total lipid fraction (TL) extracted and purified from CS on the proliferation and cytokine profile of sheep PBMCs.

## 2. Materials and Methods

### 2.1. Microalgae Cultivation

An outdoor closed vertical tubular photobioreactor (PBR) (400 L volume) (Aqualgae SL, Almerìa, Spain) was used for phototrophic cultivation of the monoxenic strain of CS (UTEX 2805) as previously reported in Morgese et al. [12]. The algal biomass obtained was freeze-dried and stored at −20 °C.

### 2.2. Microalgal Extract Preparation and Chemical Characterization

Lipids from freeze-dried algal biomass of CS were extracted as previously reported by Francavilla et al. [13]. The acetylation of the AUP was performed using acetic anhydride (Ac_2_O) and anhydrous NiCl_2_ as a catalyst under solvent free-conditions according to Meshram and Patil [14]. 

The hydroalcoholic residue of the UP extraction process was separated in order to collect methylated fatty acids (FAMEs) according to Morgese et al. [12].

### 2.3. Animals and Experimental Treatments

PBMCs were collected from the blood of 20 healthy dairy sheep balanced for age, sex, body condition score (BCS), and parity by density gradient centrifugation according to Wattegedera et al. [15]. Animals were located at the Segezia research station of the Council for Research and Experimentation in Agriculture. A final concentration of 2 × 10 ^5^ cells/mL in Iscove’s Modified Dulbecco’s medium (IMDM) (Sigma Aldrich, Milan, Italy) containing 10% fetal bovine serum (FBS) (Sigma Aldrich, Milan, Italy) and 50 μg/mL gentamicin (Sigma Aldrich, Milan, Italy) was seeded into a 96-well U-bottom plate (Sigma Aldrich, Milan, Italy).

### 2.4. PBMCs for Lymphocyte Stimulation Assay and Cytokine Determination

PBMCs were treated with the UP, the AUP, and the TL fractions extracted and purified from CS. Cells were stimulated with concanavalin A (ConA, at a final concentration of 5 μg/mL) and lipopolysaccharide (LPS, at a final concentration of 1 μg/mL). For each extract, two different concentrations were tested (0.4 mg/mL and 0.8 mg/mL). PBMCs cultured without ConA and LPS represented the unstimulated cells (USC). PBMCs cultured with ConA and LPS represented the stimulated cells (SC). The plates were incubated at 37 °C and 5% CO_2_ in a humidified incubator for 24 h. After the incubation time, the plates were centrifuged, and the cell-free supernatants from each well were collected and stored at −20 °C until the ELISA to measure cytokine production.

On cells, a proliferation test was performed based on the incorporation of bromodeoxyuridine (BrDU) in dividing cells using a commercial kit (Roche) (Sigma Aldrich, Milan, Italy); after 18 h of incubation, the BrDU incorporated during DNA synthesis was measured by reading the optical density with a plate reader spectrophotometer (Power Wave XS, Biotek, UK) at 450 nm.

### 2.5. Determination of Cytokines in Culture Supernatant by ELISA

The ELISA for IL-6, IL-1β, and IL-10 in cell-free supernatants was determined according to Ciliberti et al. [10]. Briefly, for IL-6 and IL-1β, all the incubations were at 37 °C, whereas for IL-10, they were at room temperature. Each cytokine quantification was read in duplicate against a specific standard curve fitting a 4-parameter logistic curve. In particular, the standard curve for IL-6 was obtained using scalar dilution of recombinant ovine IL-6 (Cusabio Biotech Co., Wuhan, P.R.China), IL-1β was obtained using scalar dilution of recombinant ovine IL-1β (Kingfisher Biotech Inc., St Paul, USA), and IL-10 was obtained using scalar dilution of recombinant bovine IL-10 (Kingfisher Biotech Inc., St Paul, USA). The R^2^ values were 99.9% for IL-6, 100% for IL-1β, and 99.7% for IL-10.

### 2.6. Statistical Analysis

The normality of the variables was checked by Shapiro–Wilk test [16]. A Log transformation was performed when data were not normally distributed. Data were processed using a one-way ANOVA [17]. When significant differences between means (*p* < 0.05) were found, the Fisher’s least significant difference test was used as post-hoc test for multiple comparisons.

### 2.7. Ethical approval

The experimental design and animal procedures were carried out and approved in accordance with the Foggia University Institutional Animal Care and Use Committee (protocol number 0002302).

## 3. Results

### 3.1. Chemical Characterization of Microalgal Extracts 

**Figure 1:**
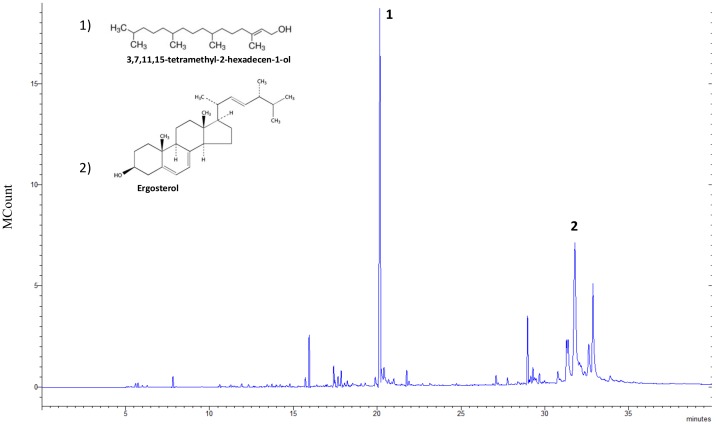
Total ion chromatogram of the unsaponified fraction (UP) of *Chlorella sorokiniana* and molecular structures of the predominant constituents.

**Figure 2:**
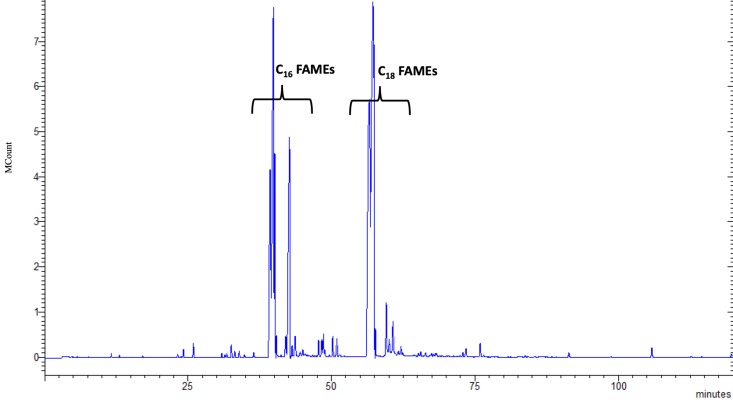
Total ion chromatograms of fatty acids methyl esters (FAMEs) from the total lipid fraction (TL) of *Chlorella sorokiniana*.

*Chlorella sorokiniana* had a total lipid (TL) content of 21% dry weight (dw) which was composed of fatty acids (42% dw TL) and an unsaponified fraction (UP) (19% dw TL), according to results previously reported [12]. The TL fraction was characterized by its two main components: the UP fraction (Figure 1) and FAMEs (Figure 2).

The most abundant UP molecules were 3,7,11,15-tetramethyl-2-hexadecen-1-ol (64.26 mg/g TL) and ergosterol (43.36 mg/g TL). Figure 2 shows the FAMEs chromatogram in which the C16 and C18 carbon chain groups represented 92% [12]. Of these, PUFAs comprised 286.7 mg/g of the TL concentration, while monounsaturated (MUFA) and saturated (SFA) fatty acids had lower concentrations. The ratio of ω-3 to ω-6 was 1.5 to 1, registering 169.5 mg/g and 114.6 mg/g of the TL concentration, respectively.

### 3.2. Proliferative Response to Clorella sorokiniana Extracts

**Figure 3:**
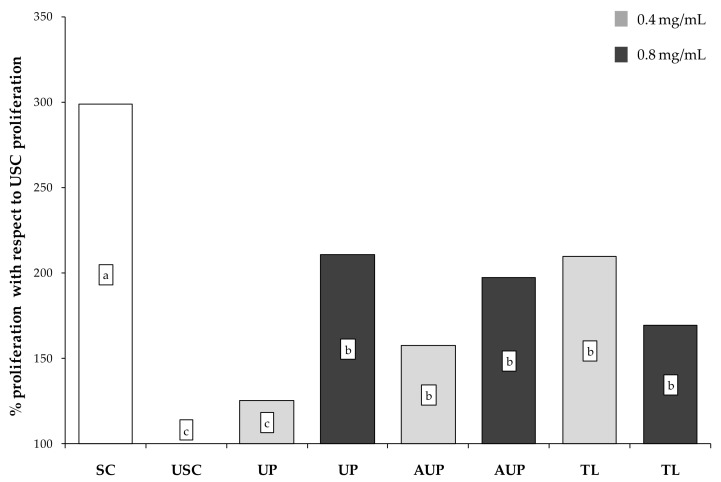
Proliferation of sheep peripheral blood mononuclear cells (PBMCs) stimulated with concanavalin A (ConA) and lipopolysaccharide (LPS) and cultured with the unsaponified fraction (UP), the acetylated unsaponified fraction (AUP), and the total lipids fraction (TL) extracted and purified from *C. sorokiniana*. For each treatment, 0.4 mg/mL and 0.8 mg/mL were tested on PBMCs. PBMCs with ConA and LPS were stimulated cells (SC), and PBMCs without ConA and LPS were unstimulated cells (USC). Data were analyzed by one-way ANOVA and Fisher post-hoc test and are presented as % proliferation exerted by each tested extract with respect to USC proliferation (100%). a, b, c indicate *p* < 0.05.

Data on proliferation are represented as percentages of PBMC proliferation calculated with respect to USC proliferation, which was considered as 100% (Figure 3). The addition of ConA and LPS to PBMCs resulted in a significant increase in cell proliferation as shown by the comparison between SC and USC proliferation. The CS extracts affected PBMC proliferation (*p* < 0.001). All CS extracts reduced PBMC proliferation in response to stimulation with ConA and LPS as demonstrated by comparison with the proliferation of SC. The UP extract at 0.4 mg/mL resulted in the lowest proliferation in comparison to all the other extracts, showing the same proliferation level as PBMCs in the absence of stimulation (USC). Moreover, the PBMC proliferation in response to UP extract was dose-dependent because the proliferation at 0.4 mg/mL concentration was lower than that at 0.8 mg/mL.

### 3.3. Cytokine Production by PBMCs

**Figure 4:**
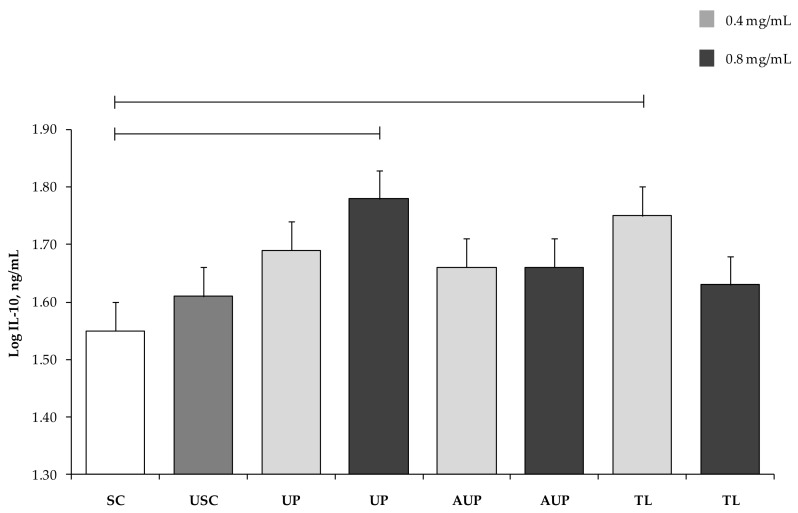
Interleukin (IL)-10 secretion by PBMCs stimulated with ConA and LPS and cultured with the unsaponified fraction (UP), the acetylated unsaponified fraction (AUP), and the total lipids fraction (TL) extracted and purified from *C. sorokiniana*. For each treatment, 0.4 mg/mL and 0.8 mg/mL were tested on PBMCs. PBMCs with ConA and LPS were stimulated cells (SC), and PBMCs without ConA and LPS were unstimulated cells (USC). Data were analyzed by one-way ANOVA and Fisher post-hoc test and are presented as least squares mean ± SEM. Bars indicate *p* < 0.05.

IL-10 production in PBMCs stimulated with ConA and LPS was not different from that in USC (Figure 4). On the contrary, PBMCs cultured with 0.8 mg/mL UP and with 0.4 mg/mL TL exhibited higher IL-10 production than PBMCs stimulated with ConA and LPS (*p* < 0.05). 

**Figure 5:**
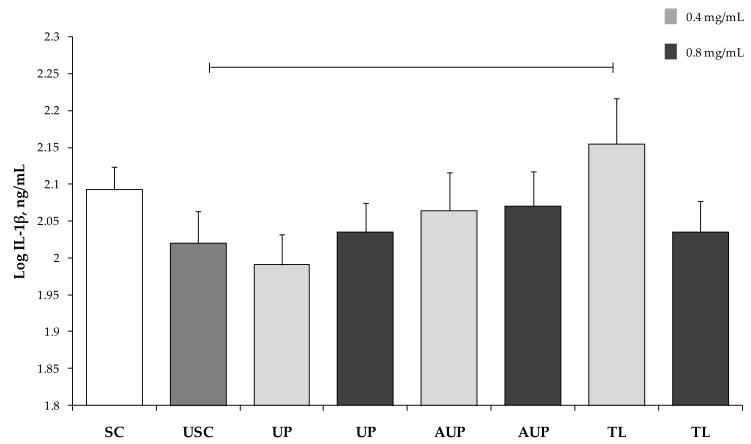
Interleukin (IL)-1β secretion by PBMCs stimulated with ConA and LPS and cultured with the unsaponified fraction (UP), the acetylated unsaponified fraction (AUP), and the total lipids fraction (TL) extracted and purified from *C. sorokiniana*. For each treatment, 0.4 mg/mL and 0.8 mg/mL were tested on PBMCs. PBMCs with ConA and LPS were stimulated cells (SC), and PBMCs without ConA and LPS were unstimulated cells (USC). Data were analyzed by one-way ANOVA and Fisher post-hoc test and are presented as least squares mean ± SEM. Bar indicates *p* < 0.05.

**Figure 6:**
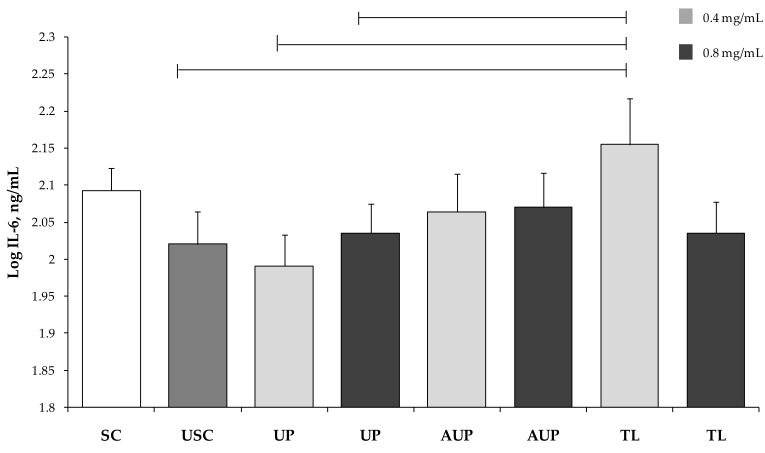
Interleukin (IL)-6 secretion by PBMCs stimulated with ConA and LPS and cultured with the unsaponified fraction (UP), the acetylated unsaponified fraction (AUP), and the total lipids fraction (TL) extracted and purified from *C. sorokiniana*. For each treatment, 0.4 mg/mL and 0.8 mg/mL were tested on PBMCs. PBMCs with ConA and LPS were stimulated cells (SC), and PBMCs without ConA and LPS were unstimulated cells (USC). Data were analyzed by one-way ANOVA and Fisher post-hoc test and are presented as least squares mean ± SEM. Bars indicate *p* < 0.05.

Both IL-1β and IL-6 were higher in PBMCs cultured with 0.4 mg/mL TL versus unstimulated PBMCs (*p* < 0.05; Figure 5 and Figure 6). In addition, the IL-6 produced by PBMCs with 0.4 mg/mL TL was higher than IL-6 from PBMCs with both concentrations of UP (*p* < 0.05). 

## 4. Discussion

This experiment studied the in vitro immunological effects of *C. sorokiniana* extracts on sheep cells. Animal health is intimately connected to their feed, and higher quality of feed can improve their survival, growth, development, productivity, and fertility [2]. Recently, extracts from *Chlorella* have been proposed as a food to improve human health [18]; they have been successfully applied to modulate the human immune response and relieve hypertension [19,20,21,22].

*Chlorella* can also promote the growth rate of animals [23], boost their immune function [24], accelerate dioxin elimination [25], prevent stress-induced ulcers [26], and markedly decrease high-fat-diet-induced dyslipidemia [27]. Accordingly, our data demonstrated an anti-proliferative effect of CS extracts on sheep PBMCs, and this was particularly apparent when cells were treated with UP extracts at lower concentrations. Chemical characterization of the CS extract showed that the most abundant molecules of the UP extract were 3,7,11,15-tetramethyl-2-hexadecen-1-ol and ergosterol. Recently, the anti-proliferative role of the two main phytosterols (7-dehydroporiferasterol and ergosterol) extracted and purified from *D. tertiolecta* was thoroughly studied on sheep PBMCs [11]. Similar inhibitory results of phytosterols from *D. tertiolecta* in sheep PBMCs during the postpartum period were found [10]. The immunological effects of phytosterols on cell proliferation are associated with their chemical structure; in particular, sterol components can relieve chronic gastric ulcers in a rat model [28], a mouse skin cancer model, and HT29 colon adenocarcinoma [29,30]. The strong nexus between the molecular structures of phytosterols and their anti-proliferative effects was observed mainly in the UP fraction. The acetylated UP fraction did not have the same consistent reduction in cell proliferation as the UP fraction. The TL fraction was a mixture of the UP and the saponified fraction; however, we did not observe a synergistic action on PBMC proliferation. These results underscore the need to understand the role exerted by each molecule in CS extracts on immunological outcomes. More recently, Lin et al. [31] showed the possible molecular mechanism governing the anti-tumor activity of CS on non-small cell lung cancer cell lines (NSCLC). This activity seems to be correlated to caspase mitochondrial dysfunction and down-regulated XIAP and survinin, with both of these increasing NSCLC apoptosis. The anti-proliferative role of the CS extracts is likely orchestrated by increasing the susceptibility of PBMCs to apoptosis.

The spectra of biological responses activated by the alga *Chlorella* are also a direct modulation of cytokine production [32,33,34,35]. As a result, both IL-6 and IL-1β levels increased in PBMCs treated with the TL fraction. This suggests that it can restore homeostasis when acting on an immunosuppression status. Dairy sheep are frequently immunosuppressed before partum and after exposure to high temperatures [36,37]. In these contexts, the animals have increased susceptibility to invasive pathogens; therefore, the physiological state of stress could be pathological. Treatment with nutraceutical products such as *C. sorokiniana* might benefit animal health in addition to having high nutritional values. We found that 0.4 mg/mL TL increased the production of proinflammatory cytokines and restored PBMC competence to produce IL-10 as a mediator of the inflammatory response. Moreover, the IL-10 increased in the presence of CS extracts, while SC and USC treatment did not result in significant differences in IL-10 production. Thus, the effects of the CS fractions were an additive effect to ConA and LPS stimulation as regards IL-10 secretion by PBMCs. This additive effect on IL-10 secretion of the CS extracts should be highlighted considering that IL-10 is produced as the main regulatory factor in pro-inflammatory status and it can drive the time and intensity of the inflammatory response [15]. The TL fraction was rich in fatty acids, especially ω-3 (169.53 mg/g of TL) and ω-6 (116.66 mg/g of TL), as well as in molecules typical of the UP fraction. Interestingly, bovine endothelial cells with enhanced ω-3 phospholipid contents have reduced inflammatory responses [38], the increased level of ω-3 fatty acids in the membrane phospholipid promoting the synthesis of inflammation-resolving eicosanoids [38]. Sheep fed flaxseed, rich in α-linolenic (ALA), exhibited increased IL-10 and IL-6 postpartum. This mitigates excessive inflammation [39]. Moreover, PBMCs from sheep fed a diet rich in ω-3 and subsequently treated in vitro with phytosterols from *D. tertiolecta* had a main role exerted by ω-3 in driving cytokine production [10]. Conversely, sheep PBMCs treated with a mixture of ergosterol and 7-dehydroporiferasterol from *D. tertiolecta* had decreasing levels of IL-6 and TNF-α and an increased level of IL-10. The importance of the various degrees of simultaneous expression of pro- and anti-inflammatory cytokines in bovine monocytes was reported and shown to resolve uterine infections [40]. Accordingly, our experiment shows simultaneous augmentation of both pro-inflammatory and anti-inflammatory cytokine levels via TL extract, which can be considered a feed ingredient useful for resolving inflammatory conditions. The molecular characterization of UP extract showed the presence of 3,7,11,15-tetramethyl-2-hexadecen-1-ol; this molecule is associated with the activation of the peroxisome proliferator-activated receptors α (PPAR)—a transcription factor that is constitutively expressed in all cell tissues [41]. This activation increased the expression of several peroxisomal and mitochondrial oxidation enzymes, leading to changes in fatty acid metabolism. Furthermore, PPARα exerted an anti-inflammatory effect by binding to the response element of nuclear factor kappa B (NFκB) and blocking the transcription of inflammatory genes [41]. These findings help to explain the anti-inflammatory effect of UP extract by increasing the IL-10 production as a possible result of the activation of PPARα gene expression mediated by 3,7,11,15-tetramethyl-2-hexadecen-1-ol. Such IL-10 increase leads to an immunosuppressive phenotype in cells and higher resistance to stressors as reported by Spaner et al. [42]. Furthermore, IL-10 levels registered in PBMCs treated with 0.8 mg/mL UP extract are likely associated with increased proliferation versus the proliferation observed when UP extract was used at lower concentrations. The concomitant UP-dose-dependent increase in cell proliferation and IL-10 production could be indicative of a shift to Th1 response. Likewise, the possibility of inducing or inhibiting a clear degree of responsiveness might have a key impact on any immune response targeting an encountered pathogen [43]. Finally, the capability to recover a normal pattern of cytokines exploited by CS microalga could be the basis of its adaptogenic mechanism in a sheep model as shown in previous human studies [32,33,34,35]. 

## 5. Conclusions

In summary, this study demonstrated the ability of CS extracts to affect sheep PBMC proliferation and cytokine production. These effects were according to the chemical profile of the molecules characterizing each extract. The 0.4 mg/mL UP was the strongest inhibitor of PBMC proliferation; it also showed an increase in PBMC proliferation at 0.8 mg/mL. Furthermore, the UP extract at higher concentrations was also characterized by increased IL-10 production, which could possibly be attributed to the presence of 3,7,11,15-tetramethyl-2-hexadecen-1-ol. The UP fraction could reduce the level of inflammation during inflammatory states like postpartum. 

Conversely, the TL fraction at 0.4 mg/mL showed a cytokine profile characterized by increasing IL-10, IL-6, and IL-1β (to a lesser extent). This is probably due to the synergistic action exerted by sterols and fatty acids.

*Chlorella sorokiniana* extracts modulate sheep in vitro immune responses and can be a candidate ingredient in animal feeds to restore immune competence after partum or heat stress. Nutritional strategies are crucial to control inflammatory states in order to avoid the damage caused by excessive inflammation.
